# Temporalis muscle thickness as an indicator of sarcopenia predicts progression-free survival in head and neck squamous cell carcinoma

**DOI:** 10.1038/s41598-021-99201-3

**Published:** 2021-10-05

**Authors:** Boeun Lee, Yun Jung Bae, Woo-Jin Jeong, Hyojin Kim, Byung Se Choi, Jae Hyoung Kim

**Affiliations:** 1 Department of Radiology, College of Medicine, Ewha Womans University, Ewha Womans University Seoul Hospital, 260, Gonghang-daero, Gangseo-gu, Seoul, 07804 Republic of Korea; 2 Department of Radiology, Seoul National University Bundang Hospital, 82, Gumi-ro 173beon-gil, Bundang-gu, Seongnam, 13620 Republic of Korea; 3 Department of Otolaryngology-Head and Neck Surgery, Seoul National University Bundang Hospital, 82, Gumi-ro 173beon-gil, Bundang-gu, Seongnam, Republic of Korea; 4 Department of Pathology, Seoul National University Bundang Hospital, 82, Gumi-ro 173beon-gil, Bundang-gu, Seongnam, Republic of Korea

**Keywords:** Cancer, Diseases, Medical research, Oncology

## Abstract

Temporalis muscle thickness (TMT) on brain magnetic resonance imaging (MRI) is correlated with sarcopenia and can be a predictive marker for survival in patients with brain tumors, but the association of TMT on head and neck computed tomography (CT) with survival in head and neck squamous cell carcinoma (HNSCC) remains unclear. We investigated whether TMT on CT could predict progression-free survival (PFS) in patients with HNSCC. A total of 106 patients with newly diagnosed HNSCC were included in this retrospective study. The patients underwent baseline head and neck CT and/or MRI between July, 2008 and August, 2018. The correlation between TMT on CT and MRI was tested using intraclass correlation coefficient (ICC). The cut-off value of TMT on CT for determining tumor progression was identified using receiver-operating characteristic curve analysis. Uni- and consecutive multi-variable Cox regression models were used to verify the association between TMT and PFS. TMT on CT and MRI showed excellent correlation (ICC, 0.894). After a mean follow-up of 37 months, 49 out of 106 patients showed locoregional recurrence and/or distant metastasis. The cut-off TMT of 6.47 mm showed good performance in predicting tumor progression (area under the curve, 0.779). The Cox regression model showed that TMT ≤ 6.24 mm (median value in study population) was a significant contributing factor for predicting shorter PFS (hazard ratio 0.399; 95% confidence interval 0.209–0.763; *P* = .005). TMT may be used as a surrogate parameter for pre-treatment sarcopenia and could help predict PFS in patients with HNSCC.

## Introduction

Sarcopenia is defined as a progressive and generalized loss of the mass and function of the skeletal muscle and has been shown to be a negative predictor in various types of cancers^1^. In general, sarcopenia is associated with decreased survival rate, longer hospital stays, and even higher risk of postoperative complications after cancer treatment^1–6^. In head and neck squamous cell carcinoma (HNSCC), sarcopenia has been shown to act as a strong negative prognostic factor for overall survival after radiation therapy or surgery, chemotherapy-related toxicity, and wound complications after surgery^1,7–12^.

To evaluate the presence of sarcopenia in HNSCC patients, most of the previous studies have used computed tomography (CT) as a diagnostic tool^13–15^. Total body skeletal muscle mass can be estimated by measuring the cross-sectional skeletal muscle area at the level of the third lumbar vertebra (L3) or third cervical vertebra (C3)^1,13,16^. However, in patients with HNSCC, abdominal CT scans covering the L3 level are seldom available because of the additional radiation exposure and costs^13,14,17^. In addition, measuring the cross-sectional skeletal muscle area on axial CT scan by manual or semi-automated segmentation is too complex to be routinely performed in the clinical setting^1,16^.

On the other hand, in patients with brain metastases, a strong correlation has been shown between the temporal muscle thickness (TMT) measured on axial slices of brain magnetic resonance imaging (MRI) and the skeletal muscle mass measured on cross-sectional abdominal CT, enabling TMT as a surrogate marker for sarcopenia^18^. Indeed, several studies have shown that TMT can predict survival in patients with brain metastases and high-grade gliomas^18–21^. However, the association of TMT with survival prognosis, as an indicator of sarcopenia, in patients with HNSCC remains to be determined.

Therefore, in this study, we aimed to evaluate whether TMT on axial head and neck CT could be correlated with that on MRI, enabling the estimation of sarcopenia, and, more importantly, to investigate the prognostic relevance of TMT on CT in predicting tumor progression in patients with newly diagnosed HNSCC.

## Materials and methods

The institutional review board in our institution approved this retrospective study (Seoul National University Bundang Hospital, No. B-2007-627-105), and the requirement for informed consent was waived. The methods and reporting of results are in accordance with the STROBE (Strengthening the Reporting of Observational Studies in Epidemiology) guidelines.

### Study population

**Figure 1:**
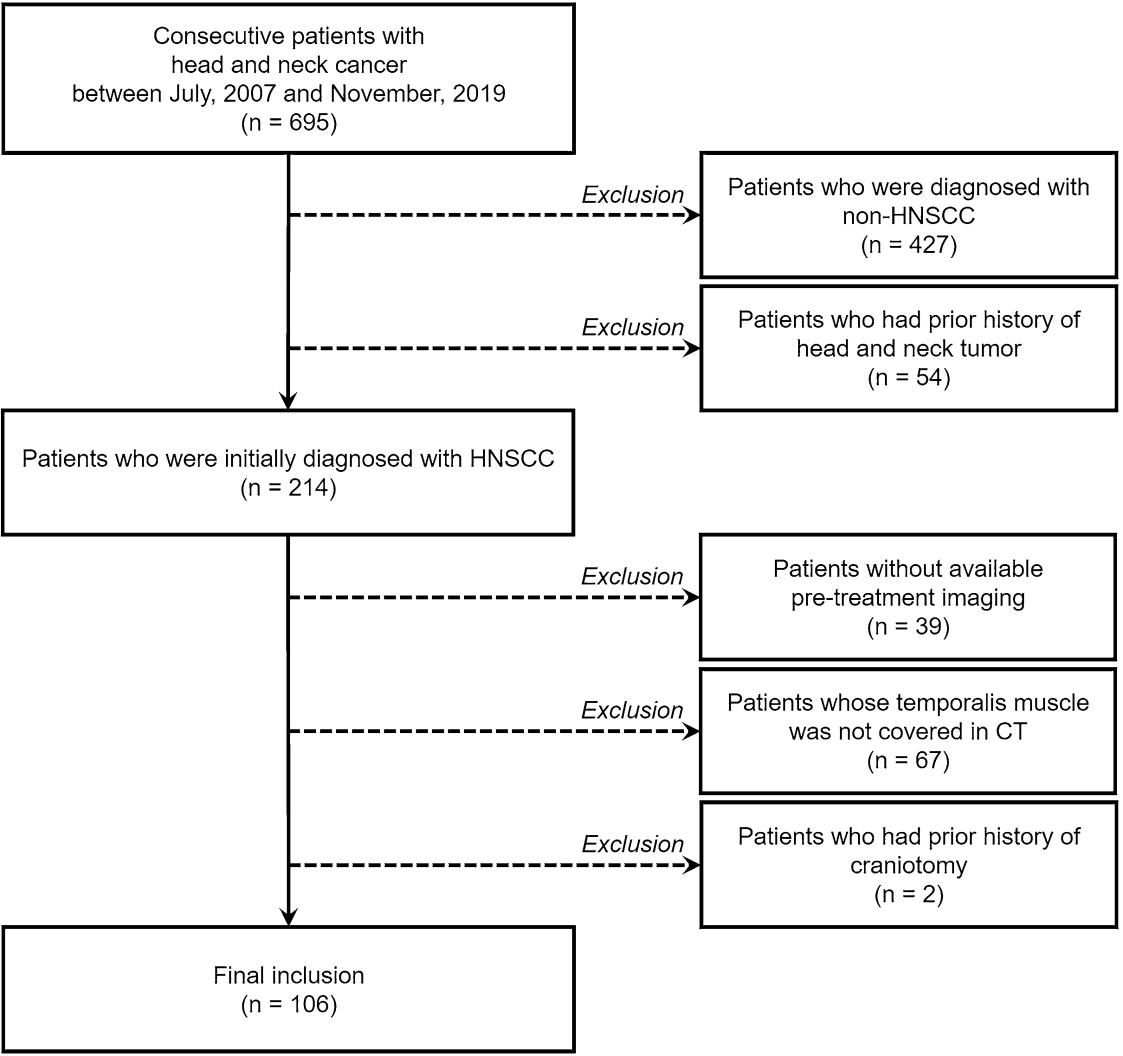
Flow diagram of the study population. *HNSCC* head and neck squamous cell carcinoma, *CT* computed tomography.

The study population was searched from the database in our tertiary referral hospital, and consecutive patients with head and neck cancer were identified between July, 2007 and November, 2019. Among them, patients were included if they were newly diagnosed with HNSCC with pathologic confirmation by biopsy or surgical excision, underwent cross-sectional head and neck CT and/or MRI before the surgery or initiation of the chemoradiation treatment, and had both sides of temporalis muscles covered in the pre-treatment cross-sectional imaging. The exclusion criteria were as follows: non-SCC diagnosis in the head and neck; previous history of head and neck cancer; previous interventions that might have affected the muscle mass such as craniotomy; no available pre-treatment cross-sectional imaging; temporalis muscle not covered in the cross-sectional CT. The flow diagram of patients’ enrollment is provided in Fig. 1. Patients’ age, sex, smoking status, tumor subsites, human papillomavirus status, clinical staging (including tumor [cT] category, node [cN] category, metastasis [cM] category, and overall stage) according to the 8th edition of the American Joint Committee on Cancer staging manual^22^, treatment modality (initial surgery, surgery after neoadjuvant chemoradiation, and adjuvant radiation or chemoradiation), and preoperative serum albumin levels were obtained from the electronic medical records. Date of death or last follow-up was collected, and progression-free survival (PFS) was defined as the time between initial diagnosis and clinical tumor recurrence or distant metastasis or the last follow-up, and overall survival (OS) was defined as the time between initial diagnosis and the death or the last follow-up.

### CT and MRI examinations

All patients underwent contrast-enhanced head and neck CT scans on 64- or 256-channel scanners (Brilliance, the IQon, and the iCT, Philips Healthcare, Best, The Netherlands). The imaging parameters were as follows: tube voltage, 120 kV; effective tube current, 200 mAs; collimation, 64 × 0.625; pitch, 0.829; rotation time, 0.5; raw slice thickness/increment, 2/1 mm; axial reconstruction slice thickness/increment, 3/3 mm. The scan range extended from the upper margin of the frontal sinus to the carina. For contrast-enhancement, a single-phase bolus injection of 100 cc of 350 mg/mL iodinated contrast media (Iomeron 350 mg/mL, Bracco, UK) was administered at a flow rate of 2 cc/s, and image acquisition began 80 s after the injection.

MRI was performed using a 3-T MR scanner (Achieva, Ingenia and Ingenia CX; Philips Medical Systems, Best, The Netherlands) with a 16- or 32-channel sensitivity encoding head coil. The head and neck MRI protocol contained the following sequences: axial T1-weighted image (WI); axial T2-WI with/without fat suppression; coronal T2-WI with fat suppression; and gadolinium-enhanced T1-WI in axial, coronal, and sagittal planes. Axial contrast-enhanced T1-WI was obtained after intravenous administration of a bolus of gadolinium-based contrast agent (gadobutrol, Gadovist^®^, 0.1 mmol/kg; Bayer Healthcare, Berlin, Germany) using the following parameters: repetition time, 600 ms; echo time, 15 ms; field of view, 180 × 220 mm; acquisition matrix, 440 × 440; slice thickness, 3 mm; no slice gap; number of excitations, 1. All radiological images were exported to the PACS system and analyzed.

### TMT measurement

**Figure 2:**
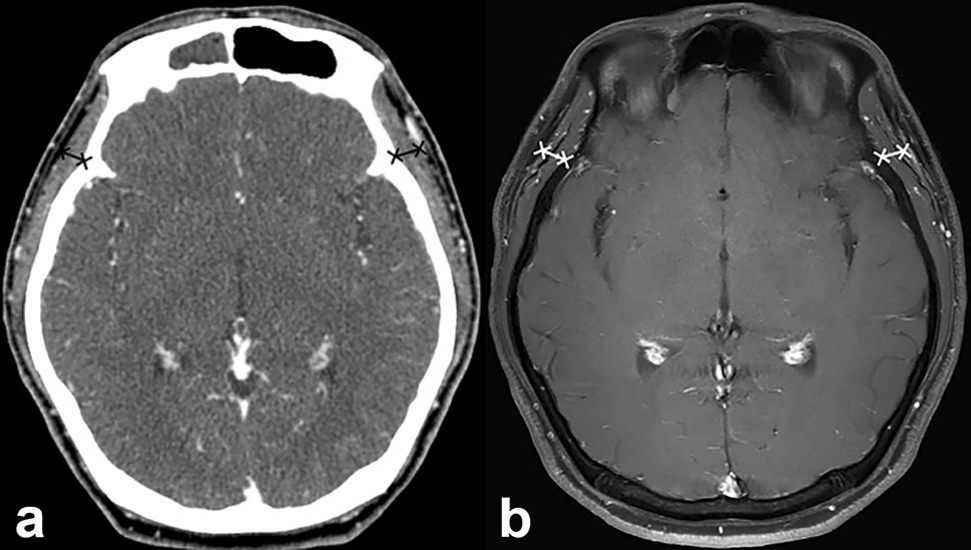
Representative case for the assessment of temporalis muscle thickness (TMT) on CT and MRI. A 41-year-old patient has an averaged TMT of 6.66 mm on CT (**a**) and 8.04 mm on MRI (**b**).

All CT and/or MRI examinations were independently interpreted by two board-certified radiologists (Y.J.B. and B.S.C, with 10 and 20 years of clinical experience in head and neck imaging, respectively). TMT was measured using previously described methods^19^. On axial post-contrast CT and T1-WI of MRI, TMT was measured on the right and left sides separately in all patients, perpendicular to the long axis of the temporalis muscle, using the Sylvian fissure as reference (Fig. 2). Then, the averaged values were used for further analysis.

### Statistical analysis

Continuous variables were expressed as mean ± standard deviation. By setting the median value of the TMT as a reference^19,20^, all patients were categorized into two groups. The patient’s clinicopathological features were compared between the groups using a chi-square test or Fisher’s exact test for categorical variables and a Student’s t test for continuous variables.

The correlation of the TMT measurements between two readers was tested using the interclass correlation coefficient (ICC). Then, the correlation between the averaged TMT measured on CT and that measured on MRI was tested using ICC and the Pearson correlation coefficient. The values of ICC were interpreted using the following criteria: poor agreement for values < 0.40; fair agreement for 0.40–0.59; good for 0.60–0.74; and excellent for 0.75–1.00^23,24^.

To analyze the association between TMT and tumor progression, first, the diagnostic performance predicting tumor progression using TMT on CT was evaluated using receiver operating characteristic curve analysis. Next, univariable Cox regression analysis was performed to identify predictors of PFS and OS among the clinical and radiological variables. Here, the cut-off value of TMT on CT for the dichotomization of patients was also determined as the median value of TMT, as in previous publications^18–20^. Lastly, all variables with *P* values < 0.05 in univariable analysis were included in the consecutive multivariable Cox regression analysis for the estimation of the hazard ratio for predicting PFS and OS. Survival curves were estimated using the Kaplan–Meier method and compared with a log-rank test.

*P* values less than 0.05 were considered statistically significant. Statistical analyses were performed using SPSS software (version 17.0; SPSS, Chicago, IL, USA), MedCalc 17.9 (MedCalc, Mariakerke, Belgium), SAS version 9.3 (SAS Institute, Cary, NC, USA), and R version 3.5.2 (R Project for Statistical Computing, http://www.r-project.org).

## Results

### Patient characteristics

Altogether, 106 patients (22 women and 84 men; mean age, 66.4 years) with newly diagnosed HNSCC were included. All patients underwent baseline cross-sectional CT and/or MRI between July, 2008 and August, 2018. The last follow-up period was April 2020. The mean TMT in all patients was 6.25 ± 1.68 mm (range 3.10–11.09 mm) on CT, and 7.94 ± 1.75 mm (range 4.78–12.43 mm) on MRI. Age was significantly higher in patients whose TMT on CT was thicker than the median value of 6.24 mm than in patients with TMT ≤ 6.24 mm (*P* = 0.038). There were no significant differences in clinical variables between the two patient groups dichotomized according to the median TMT on CT (Table 1).

**Table 1:** Clinical characteristics according to the TMT measurement.

	No. (%)	TMT on CT (mm)		*P *value
		> 6.24 mm	≤ 6.24 mm	
No. (%)	106 (100%)	53 (50%)	53 (50%)	NA
**Mean age (years)**		68.8 ± 2.8	64.1 ± 0.8	0.038*
**Sex**				
Men	84 (79.2%)	38 (71.7%)	46 (86.8%)	0.056
Women	22 (20.8%)	15 (28.3%)	7 (13.2%)	
**Smoking status**				
Never smoked	56 (52.8%)	31 (58.5%)	25 (47.2%)	0.195
Former smoker	26 (24.5%)	9 (17.0%)	17 (32.1%)	
Current smoker	24 (22.6%)	13 (24.5%)	11 (20.8%)	
**Treatment modality**				
Initial surgery	13 (12.3%)	7 (13.2%)	6 (11.3%)	0.739
Surgery after neoadjuvant chemoradiation	37 (34.9%)	20 (37.7 5)	17 (32.1%)	
Adjuvant radiation or chemoradiation	37 (34.9%)	20 (37.7 5)	17 (32.1%)	
**Human papillomavirus**				
Negative	79 (74.5%)	41 (77.4%)	38 (71.7%)	0.505
Positive	27 (25.5%)	12 (22.6%)	15 (28.3%)	
**Tumor site**				
Sinonasal cavity	8 (7.5%)	5 (9.4%)	3 (5.7%)	0.944
Oral cavity	17 (16.0%)	9 (17.0%)	8 (15.1%)	
Nasopharynx	14 (13.2%)	6 (11.3%)	8 (15.1%)	
Oropharynx	33 (31.1%)	17 (32.1%)	16 (30.2%)	
Hypopharynx	12 (11.3%)	5 (9.4%)	7 (13.2%)	
Larynx	22 (20.8%)	11 (20.8%)	11 (20.8%)	
**cT category**				
T1	16 (15.1%)	5 (9.4%)	11 (20.8%)	0.103
T2	31 (29.2%)	13 (24.5%)	18 (34.0%)	
T3	27 (25.5%)	18 (34.0%)	9 (17.0%)	
T4	32 (30.2%)	17 (32.1%)	15 (28.3%)	
**cN category**				
N0	43 (40.6%)	22 (41.5%)	21 (39.6%)	0.749
N1	25 (23.6%)	12 (22.6%)	13 (24.5%)	
N2	27 (25.5%)	12 (22.6%)	15 (28.3%)	
N3	11 (10.4%)	7 (13.2%)	4 (7.5%)	
**cM category**				
M0	53 (50.0%)	48 (48.5%)	5 (71.4%)	0.243
M1	53 (50.0%)	51 (51.5%)	2 (28.6%)	
**Overall stage**				
I	18 (17.0%)	6 (11.3%)	12 (22.6%)	0.383
II	20 (18.9%)	10 (18.9%)	10 (18.9%)	
III	34 (32.1%)	17 (32.1%)	17 (32.1%)	
IV	34 (32.1%)	20 (37.7%)	14 (26.4%)	
**Preoperative serum albumin (g/dL)**		4.19 ± 0.47	4.04 ± 0.56	0.134

The patients were dichotomized according to the median TMT value.

*TMT* temporalis muscle thickness, *cT* clinical tumor, *cN* clinical node, *cM* clinical metastasis, *NA* not applicable.

*P value less than 0.05 indicates statistical significance.

### Correlation between TMT measured on CT and TMT measured on MRI

The correlation between the two readers for TMT measurement was excellent on CT (ICC 0.911; 95% confidence interval [CI] 0.824–0.956) and good on MRI (ICC 0.744; 95% CI 0.534–0.868). The correlation between the average TMT measured on CT and that on MRI was excellent with ICC of 0.894 (95% CI 0.848–0.927) and Pearson correlation coefficient of 0.894 (95% CI 0.790–0.948, *P* < 0.0001).

### Diagnostic performance of TMT on CT for the tumor progression

**Figure 3:**
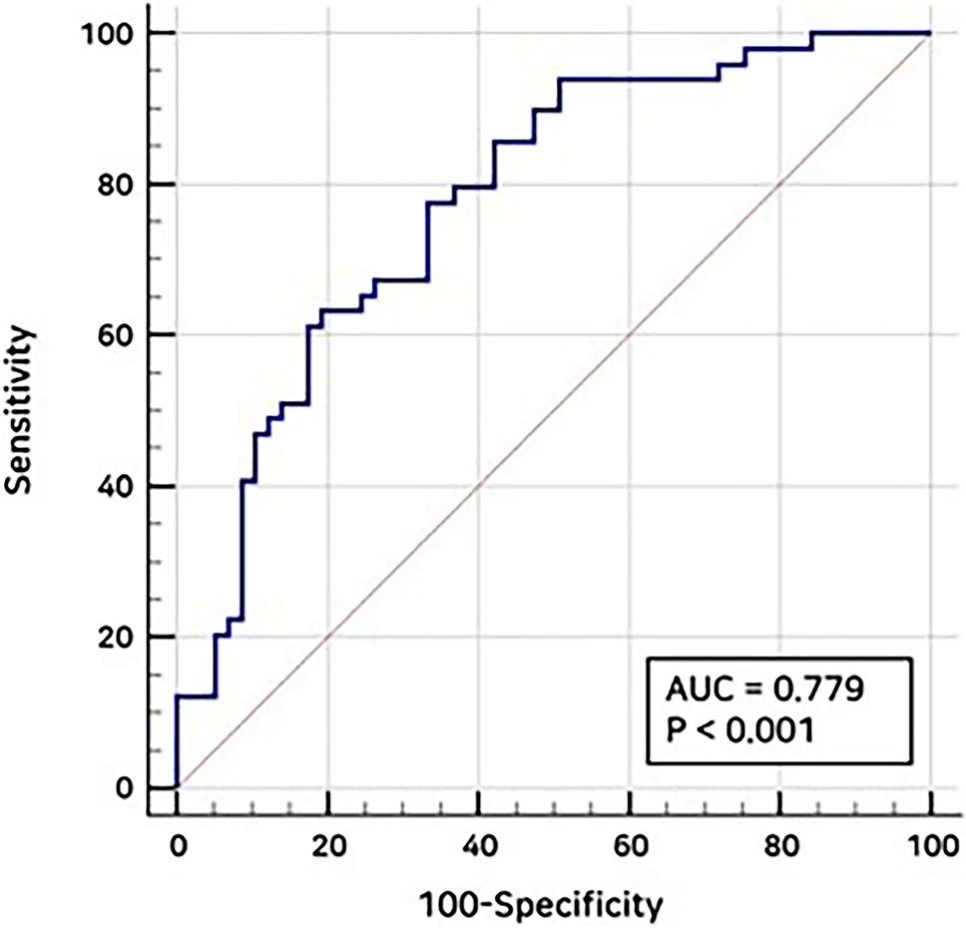
Receiver operating characteristic curve of temporalis muscle thickness (TMT) on CT for the detection of tumor recurrence. The receiver operating characteristic curve of TMT on CT shows a fair accuracy of predicting tumor recurrence with an area under the curve value of 0.779.

Forty-nine of the 106 patients showed tumor recurrence during the follow-up period. The TMT on CT was significantly lower in the tumor progression group than in the non-progression group (5.42 ± 0.37 vs. 6.96 ± 0.44, *P* < 0.0001). In the receiver operating characteristic curve analysis, TMT on CT enabled fair prediction of tumor recurrence (area under the curve, 0.779; 95% CI 0.689–0.854) (Fig. 3). Using an optimal cutoff value of 6.47 mm, a TMT < 6.47 mm showed a sensitivity and specificity of 77.5% and 66.7%, respectively, for predicting tumor recurrence.

### Survival analyses

In the univariable Cox regression analysis (Table 2), a TMT on CT thicker than the median value of 6.24 mm was a significant prognostic factor for improved PFS, with a hazard ratio of 0.358 (95% CI 0.195–0.658; *P* = 0.001). On the other hand, higher cT category (T4 vs. T1; hazard ratio, 2.667; *P* = 0.042), higher cN category (N2 vs N0 and N3 vs N0; hazard ratio, 2.406 and 5.185; *P* = 0.004 and 0.017, respectively), higher cM category (M1 vs M0; hazard ratio, 6.164; *P* < 0.0001), and higher overall stage (IV vs I; hazard ratio, 3.68; *P* = 0.009) were all significant adverse variables for PFS. Other clinical variables did not show significant association with PFS prognosis.

**Table 2:** Univariable analysis of progression-free survival.

Predictors	Hazard ratio	95% CI		*P *value
		Lower	Upper	
**TMT median**				
> 6.24 mm vs. ≤ 6.24 mm	0.358	0.195	0.658	0.0001*
**cT category**				
T2 vs. T1	0.968	0.357	2.624	0.948
T3 vs. T1	1.776	0.663	4.756	0.252
T4 vs. T1	2.666	1.037	6.855	0.041*
**cN category**				
N1 vs. N0	1.138	0.4967	2.607	0.76
N2 vs. N0	2.406	1.171	4.947	0.017*
N3 vs. N0	5.185	2.196	12.24	0.0001*
**cM category**				
M1 vs. M0	6.164	2.47	15.38	0.0001*
**Overall stage**				
II vs. I	1.031	0.3226	3.294	0.959
III vs. I	1.583	0.5682	4.409	0.38
IV vs. I	3.68	1.3897	9.742	0.009*
Age (years)	1.019	0.995	1.044	0.121
**Sex**				
Men vs. women	0.8239	0.4074	1.666	0.59
**Smoking status**				
Former vs. never	0.9747	0.489	1.943	0.942
Current vs. never	1.085	0.5412	2.173	0.819
**Initial surgery**				
Yes vs. no	0.728	0.403	1.317	0.294
**Surgery after neoadjuvant chemoradiation**				
Yes vs. no	1.38	0.544	3.502	0.498
**Adjuvant radiation or chemoradiation**				
Yes vs. No	2.463	0.763	7.957	0.132
**Human papillomavirus**				
Positive vs. negative	0.895	0.4517	1.773	0.75
**Tumor site**				
Oral cavity vs. sinonasal cavity	0.7416	0.186	2.958	0.672
Nasopharynx vs. sinonasal cavity	0.9071	0.224	3.673	0.891
Oropharynx vs. sinonasal cavity	1.2354	0.356	4.286	0.739
hypopharynx vs. sinonasal cavity	1.298	0.321	5.251	0.715
Larynx vs. sinonasal cavity	1.6772	0.472	5.96	0.424
**Preoperative serum albumin level**	0.8352	0.483	1.444	0.519

*CI* confidence interval, *TMT* temporalis muscle thickness, *cT* clinical tumor, *cN* clinical node, *cM* clinical metastasis.

*P value less than 0.05 indicates statistical significance.

Meanwhile, during the follow-up, the event of death occurred in 4 out of 106 patients. This number of events was too small to be used in the Cox regression analysis for predicting OS.

Multivariable analysis including TMT on CT, categories of cT, cN, cM, and overall stage demonstrated that TMT and cN3 were the only two significant predictors for PFS (Table 3). Patients with TMT > 6.24 mm had a significantly improved PFS (hazard ratio 0.399; 95% CI 0.209–0.763; *P* = 0.005) compared to patients with TMT ≤ 6.24 mm. In addition, cN3 over cN0 showed a significant association with an increased risk for progression with hazard ratio of 3.533 (95% CI 1.099–11.356; *P* = 0.034).

**Table 3:** Multivariable analysis of progression-free survival.

Predictor	Hazard ratio	95% CI		*P*-value
		Lower	Upper	
**TMT median**				
> 6.24 mm vs. ≤ 6.24 mm	0.399	0.209	0.7633	0.005*
**cT category**				
T2 vs. T1	1.576	0.503	4.936	0.435
T3 vs. T1	1.973	0.599	6.505	0.264
T4 vs. T1	2.236	0.7332	6.8156	0.157
**cN category**				
N1 vs. N0	1.178	0.474	2.927	0.724
N2 vs. N0	2.083	0.819	5.296	0.123
N3 vs. N0	3.533	1.099	11.356	0.034*
**cM category**				
M1 vs. M0	2.535	0.878	7.318	0.085
**Overall stage**				
II vs. I	0.729	0.193	2.745	0.639
III vs. I	0.904	0.249	3.285	0.878
IV vs. I	1.031	0.254	4.183	0.967

*CI* confidence interval, *TMT* temporalis muscle thickness, *cT* clinical tumor, *cN* clinical node, *cM* clinical metastasis.

*P value less than 0.05 indicates statistical significance.

**Figure 4:**
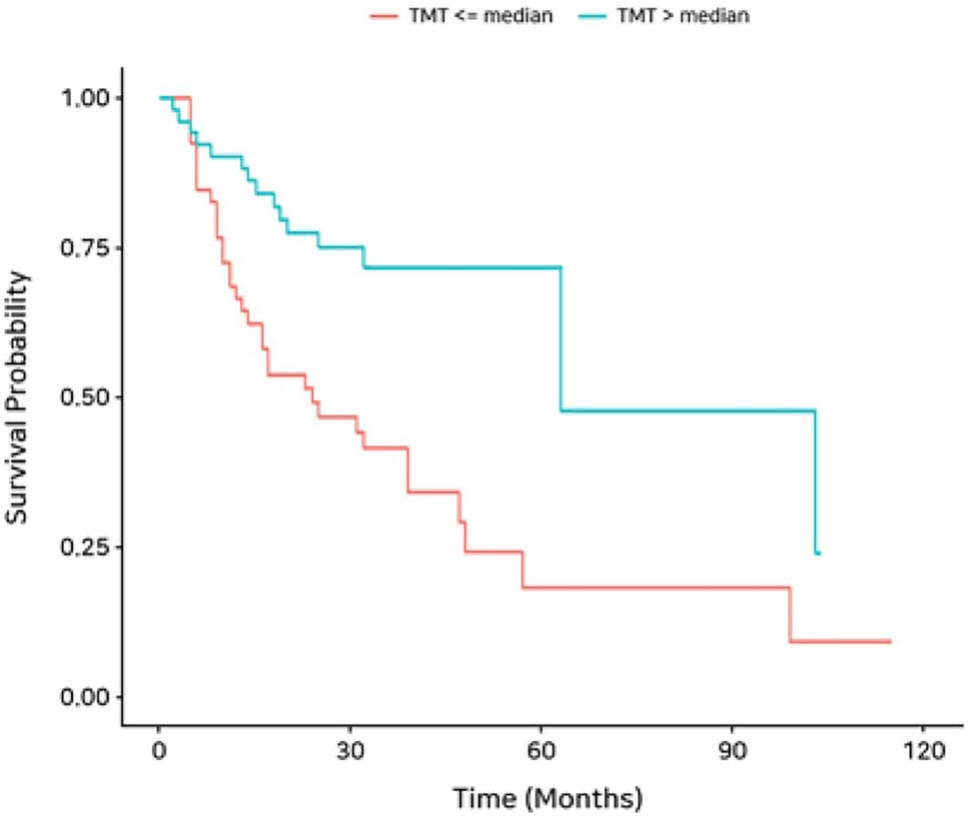
Kaplan–Meier curve for progression-free survival (PFS) according to median temporalis muscle thickness (TMT).

**Figure 5:**
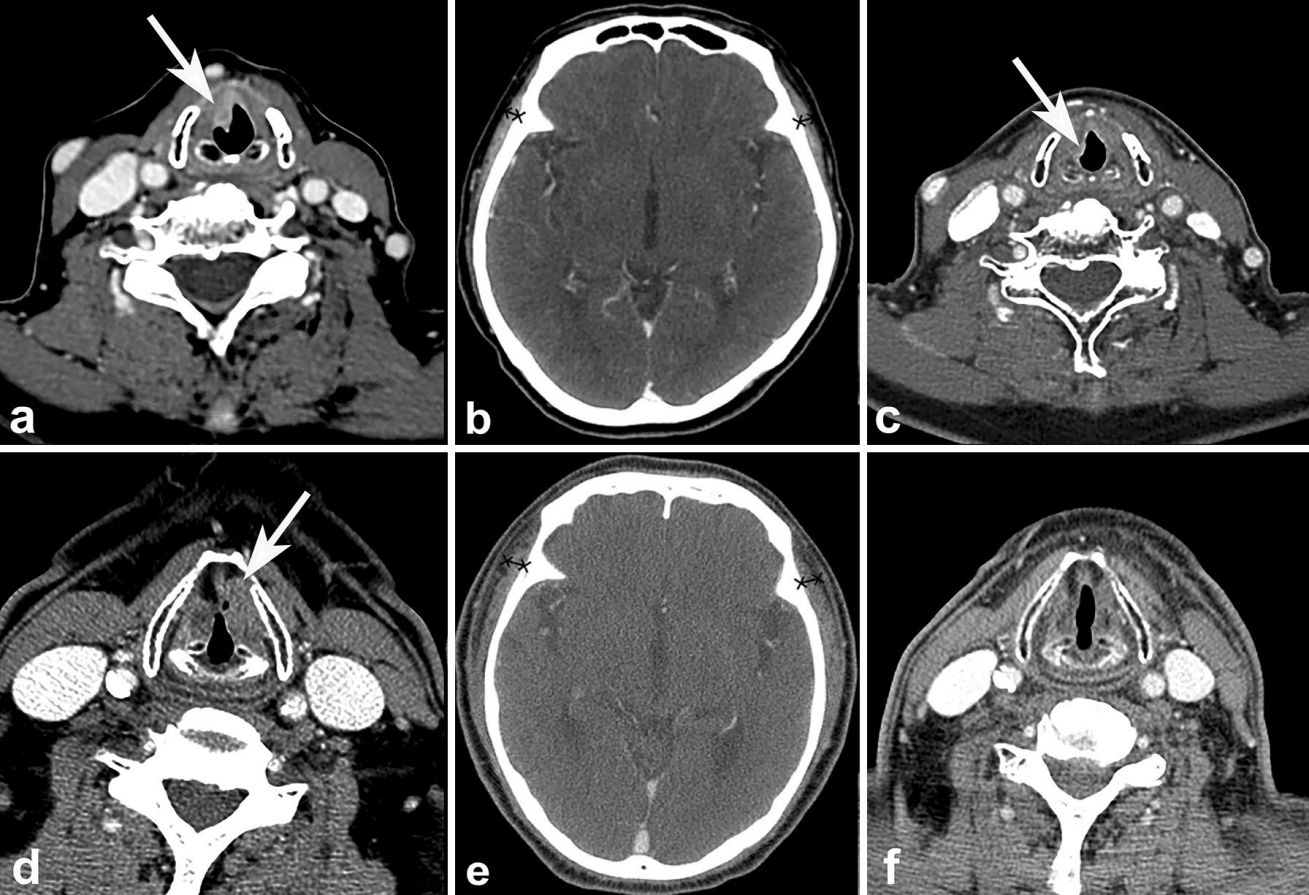
Representative cases of temporalis muscle thickness (TMT) on CT, and its association with progression-free survival. A 72-year-old female was diagnosed with glottic cancer (cT2N0M0) (**a**, arrow). On the axial CT scan, the averaged TMT was measured as 4.56 mm (**b**), which was less than the threshold of 6.24 mm. After 6 months after diagnosis, she developed local tumor recurrence in the anterior glottis (**c**, arrow). Another 74-year-old female who was diagnosed with glottic cancer (cT2N0M0) (**d**, arrow) had baseline TMT of 6.56 mm (**e**), which was larger than the threshold of 6.24 mm. During the follow-up period of 42 months, the patient did not develop tumor recurrence (**f**).

**Figure 6:**
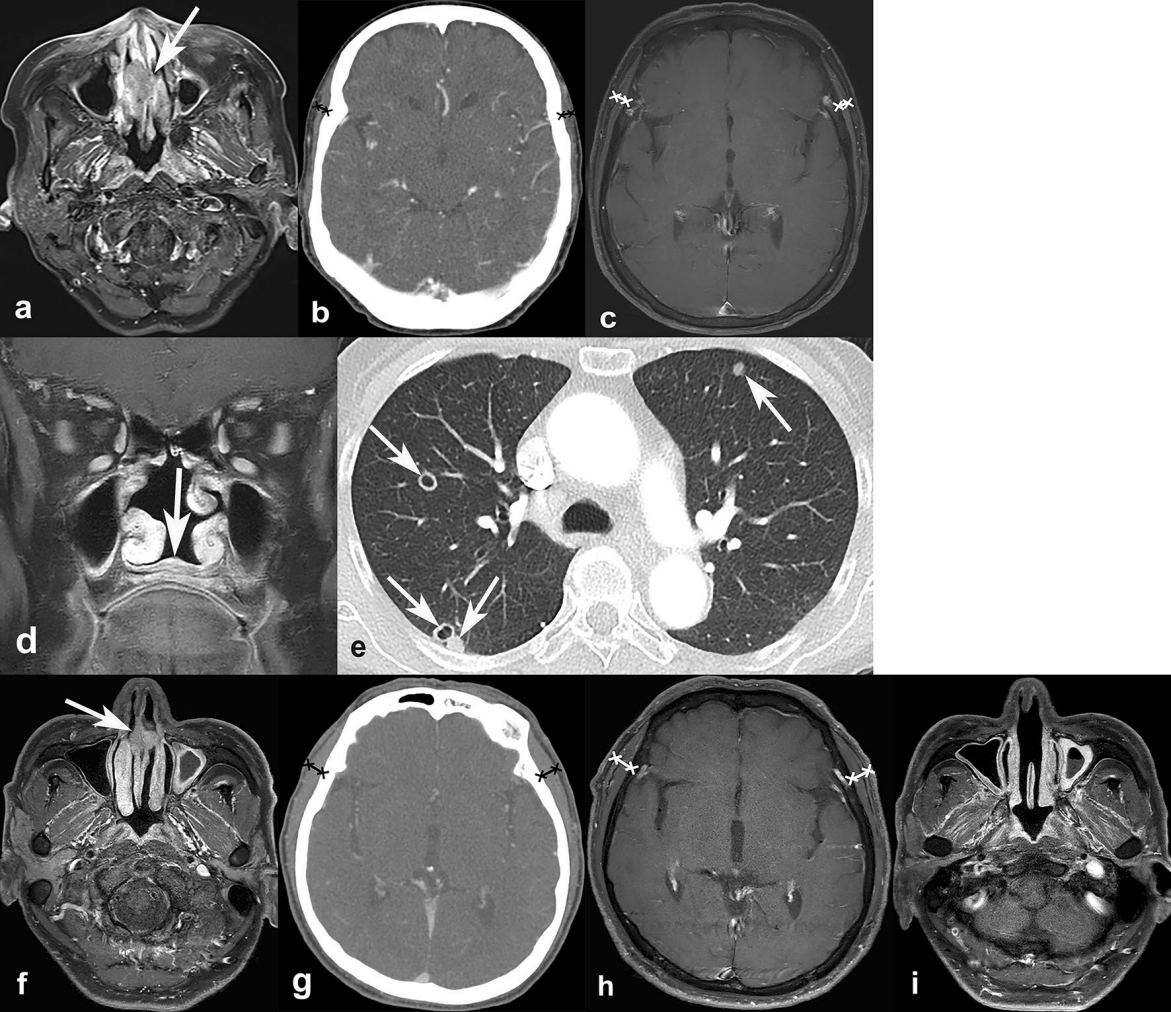
Representative cases of temporalis muscle thickness (TMT) on CT and MRI, and its association with progression-free survival. A 72-year-old female was diagnosed with nasal cavity cancer (cT2N0M0) (**a**, arrow). Her baseline TMT was measured as 4.22 mm on CT (**b**) and 4.82 mm on MRI (**c**). During the follow-up, she developed local tumor recurrence at nasal floor (**d**, arrow) and multiple pulmonary metastases (**e**, arrows). Another 66-year-old male was diagnosed with nasal cavity cancer (cT1N0M0) (**f**, arrow). His baseline TMT was 7.32 mm on CT (**g**) and 9.43 mm on MRI (**h**). After surgery, he did not show any progression during the 30 months of follow-up (**i**).

Figure 4 gives a Kaplan–Meier survival curve showing PFS according to the median value of TMT on CT. Patients with TMT > 6.24 mm presented a statistically significant longer progression-free duration (mean 68.7 months; 95% CI 50.3–87.1 months) than patients who had TMT ≤ 6.24 mm (mean, 38.5 months; 95% CI 26.2–50.6 months: log-rank *P* = 0.001) (Figs. 5, 6).

## Discussion

Although sarcopenia has been shown to act as a strong negative prognostic factor after cancer treatment, radiologic assessment of sarcopenia in pre-treatment imaging remains challenging in patients with HNSCC. In this study, we firstly demonstrated that TMT measured on axial head and neck CT can reflect the skeletal muscle mass, since it had excellent correlation with TMT on MRI which has already been proved to represent skeletal muscle mass in a previous study^18^. We also found that the TMT on CT was significantly smaller among patients with tumor progression than among patients without, enabling fair prediction of tumor progression. Most importantly, the patients with thicker pre-treatment TMT had significantly longer tumor-free survival. Thus, TMT can not only serve as an imaging surrogate parameter for sarcopenia, but also as a useful predictor for tumor progression and tumor-free survival.

Traditionally, sarcopenia has been evaluated by cervical or lumbar paravertebral muscle segmentation on serial axial CT^1,11,16,25^. Although cervical paravertebral muscle volume can be segmented on head and neck CT, this method is too complicated and time-consuming to be performed routinely. In addition, lumbar paravertebral muscles are not readily covered by head and neck CT, and additional abdominal CT scan would do more harm than good in patients with HNSCC due to the additional radiation exposure. Therefore, in clinical practice, muscle segmentation on CT to evaluate sarcopenia has rarely been implemented. In contrast, TMT measurement on a single axial slice is a simple yet effective procedure to assess sarcopenia. In addition, as shown in our study results of excellent inter-reader agreement, it can provide a consistent and reliable method to predict sarcopenia and tumor progression without additional CT scanning in HNSCC patients.

Although effective, the TMT method for predicting sarcopenia and cancer survival has not been applied other than in patients with brain tumors^18–21^. Furtner et al.^19^ assessed TMT on brain MRI in melanoma patients with brain metastases and revealed a strong correlation between the TMT measured at the sylvian fissure level and OS. Furthermore, a thicker TMT at the time of diagnosis of brain metastases was associated with a lower risk of death^19^.Similar results were obtained in patients with brain metastases from breast cancer and non-small lung cancer^20^. Most recently, the risk of lower PFS and OS with thinner baseline TMT has been verified even in patients with glioblastoma^21^. Despite the fact that sarcopenia is also an important factor in the prognosis of HNSCC^9–11^, no study has adopted TMT for assessing the presence of sarcopenia on pre-treatment head and neck CT so far. Our study was first to assess sarcopenia utilizing TMT on CT, and this may expand the role of pre-treatment imaging in routine cancer work-up from being a diagnostic tool to revealing clinically relevant prognostic factors.

It is also worth emphasizing that, after multivariable Cox regression analysis, TMT was proved to be a strongest prognostic factor for PFS, followed by the cN category. High cN and cT categories are the validated variables against good prognosis in overall cancer treatment, and positive human papillomavirus status has also been shown to be a favorable prognostic factor in oropharyngeal SCC^22^. Considering this, we can presume that TMT might become as influential as the cT and cN categories for predicting tumor recurrence-free survival in HNSCC. Such assumption should be verified in future studies with longer follow-up periods and larger study populations.

There were several limitations to this study. First, this study was performed retrospectively at a single center, potentially introducing selection biases. Future prospective studies with multi-center enrollment are needed to verify our study results. Second, our study population was heterogeneous, due to the inclusion of patients with various tumor sites, stage, and treatment methods. However, this rather could be a strength of our study, as it allowed to study HNSCC more broadly, as was done in previous studies^1,11,16^. Indeed, when we performed multivariable analysis including all clinical variables along with TMT, we could obtain significant results by Cox regression analysis. Third, due to the retrospective nature of our study, we could not obtain some clinical data that could possibly affect the treatment outcome, such as ECOG performance status, the cumulative dose of the chemotherapy agent, and the total dose of radiation therapy in the local field. Further studies that consider these clinical prognostic factors are warranted to solidify our study results. Fourth, although we focused on measuring TMT on CT, MRI can provide better soft-tissue contrast and is considered as a better imaging modality to assess primary HNSCC than CT. Thus, future study using TMT measured on MRI will add further clinical significance. Fifth, the diagnostic performance of TMT on CT in predicting tumor recurrence under receiver operating characteristic curve analysis was not very high. However, we focused on whether TMT could act as a prognostic factor for PFS, not on its diagnostic performance for tumor recurrence. Our result proved TMT on CT to be a strong significant predictor of PFS, and we believe that this result possesses greater clinical implications. Lastly, we could not perform the survival analysis for predicting OS, since the number of death events was not sufficient to be implemented in the Cox regression analysis. Therefore, further studies with longer follow-up durations are required to analyze OS and the impact of TMT on it.

In conclusion, TMT on head and neck CT may be of clinical utility as a surrogate parameter for pre-treatment sarcopenia measurement, and could help predict tumor-free prognosis in patients with HNSCC in a routine clinical setting.

## Data Availability

Data are only available upon request, and before the request, data cannot be shared publicly by the regulation of Institutional Review Board of Seoul National University Bundang Hospital, because data may contain potentially identifying or sensitive patient information. For researchers who may wish to have access to data of this study, please contact via the following e-mail and send data inquiry: msri2.snubh.org (Research Support, Institutional Review Board of Seoul National University Bundang Hospital).
